# Adaptive Attention Convolutional Neural Network for Liver Tumor Segmentation

**DOI:** 10.3389/fonc.2021.680807

**Published:** 2021-08-09

**Authors:** Shunyao Luan, Xudong Xue, Yi Ding, Wei Wei, Benpeng Zhu

**Affiliations:** ^1^Department of Optoelectronic Engineering, Huazhong University of Science and Technology, Wuhan, China; ^2^Oncology Radiotherapy Department, Hubei Cancer Hospital, Wuhan, China

**Keywords:** liver tumor, automatic segmentation, attention mechanism, CT images, deep learning

## Abstract

**Purpose:**

Accurate segmentation of liver and liver tumors is critical for radiotherapy. Liver tumor segmentation, however, remains a difficult and relevant problem in the field of medical image processing because of the various factors like complex and variable location, size, and shape of liver tumors, low contrast between tumors and normal tissues, and blurred or difficult-to-define lesion boundaries. In this paper, we proposed a neural network (S-Net) that can incorporate attention mechanisms to end-to-end segmentation of liver tumors from CT images.

**Methods:**

First, this study adopted a classical coding-decoding structure to realize end-to-end segmentation. Next, we introduced an attention mechanism between the contraction path and the expansion path so that the network could encode a longer range of semantic information in the local features and find the corresponding relationship between different channels. Then, we introduced long-hop connections between the layers of the contraction path and the expansion path, so that the semantic information extracted in both paths could be fused. Finally, the application of closed operation was used to dissipate the narrow interruptions and long, thin divide. This eliminated small cavities and produced a noise reduction effect.

**Results:**

In this paper, we used the MICCAI 2017 liver tumor segmentation (LiTS) challenge dataset, 3DIRCADb dataset and doctors’ manual contours of Hubei Cancer Hospital dataset to test the network architecture. We calculated the Dice Global (DG) score, Dice per Case (DC) score, volumetric overlap error (VOE), average symmetric surface distance (ASSD), and root mean square error (RMSE) to evaluate the accuracy of the architecture for liver tumor segmentation. The segmentation DG for tumor was found to be 0.7555, DC was 0.613, VOE was 0.413, ASSD was 1.186 and RMSE was 1.804. For a small tumor, DG was 0.3246 and DC was 0.3082. For a large tumor, DG was 0.7819 and DC was 0.7632.

**Conclusion:**

S-Net obtained more semantic information with the introduction of an attention mechanism and long jump connection. Experimental results showed that this method effectively improved the effect of tumor recognition in CT images and could be applied to assist doctors in clinical treatment.

## Introduction

Currently, liver cancer is the fifth most common malignancy and the second-leading cause of cancer-related death worldwide (1, 2). An accurate contour of the location, volume, and shape of liver tumors can help radiotherapists develop precise treatment plans. At the present time, there are several barriers to automated segmentation of liver tumors. Lesion tissue is often uniformly gray in color, which hinders automatic segmentation. Some lesions do not have clear boundaries, which limits the performance of edge segmentation methods. The specificity of lesions exists in different samples of tumors, which vary in location, size, shape, and volume. This presents further challenges to the process of segmentation. On account of these variables, automatic segmentation of tumors from the liver is a difficult task.

To solve these problems, researchers have proposed different segmentation methods, including the regional growth method, deformation model method, intensity threshold method, and the watershed algorithm. Each of these methods has individual strengths and limitations (1–9). When compared with traditional segmentation methods, fully convolutional neural networks (FCNs) have shown powerful efficacy in segmenting liver tumors. Many researchers have introduced deep learning into the liver tumor segmentation problem and found positive results.

Since U-Net was proposed by Ronneberge (10) in 2015, it has become the most common convolutional neural network architecture in medical image segmentation. Because of this finding, more U-Net derived networks were developed like the H-DenseUnet proposed by Li et al. (11) This combines U-Net with DenseNet (12) to explore the intra- and inter-slice features. U-Net++ proposed by Zhou (13) uses full-scale hopping connectivity and deep supervision to fuse high-level semantic information with low-level semantic information from feature maps at different scales and to learn hierarchical representations from multiscale aggregated feature maps. The coding-decoding network, proposed by Ginneken et al. (14), improved the accuracy of liver tumor sketching followed by shape-based post-processing to refine liver tumor margins. Roth proposed a two-stage coarse-to-fine 3D FCN. Roth HR et al. (15) proposed a two-dimensional (2D) FCN that fused three orthogonal planes to generate voxel predictions by averaging the probabilities of the three different planes.

Along with the development of different architectures of convolutional neural networks, some special modules have been proposed like the integrated attention gate (attention U-Net) by Oktay et al. (16) This network suppresses the task-irrelevant part and enhances the learning of the task-relevant part. This greatly improved the performance of semantic segmentation. Fu et al. (17) proposed DANet, a dual-attention mechanism that used network fusing channel attention and location attention to infer attention concentrated regions from two specific and mutually independent dimensions. This improved the segmentation accuracy of the model. Woo et al. (18) proposed a network called Convolutional Block Attention Module (CBAM) fusing spatial attention mechanism and channel attention mechanism. The overall architecture of the attention mechanism (19–21) is light and easy to integrate into neural networks and engage in model training end-to-end.

Although the existing algorithms have made significant achievements in liver tumor segmentation, some networks still have large and cumbersome structures. Other networks do not effectively fuse the spatial feature information captured in the down-sampling phase with the up-sampling phase. This leads to disregarding the spatial architecture of the network. To address these problems, this study proposes a small, lightweight, end-to-end, convolutional neural network of S-Net with the fusion of spatial features and attention mechanisms. The contributions of this paper include the following:

1. Proposing a pre-processing means of pixel point-to-point flipping to improve the accuracy.
2. Using small convolutional kernels and multiple batch processing to extract smaller semantic information.
3. Using a long-hop connection between the encoder and decoder to fuse spatial features and high-level semantic features.
4. Introducing attention mechanisms in neural networks to encode a longer range of semantic information in local features and to find correspondences between different channels.

**Figure 1:**
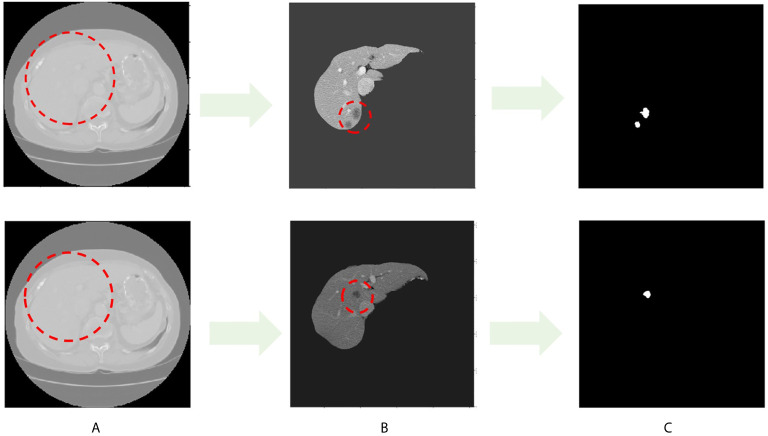
CT image sample with liver and liver tumors. **(A)** Images in LITS dataset. **(B)** Liver image. **(C)** Tumor label.

Throughout this paper, researchers used the LITS dataset, as shown in **Figure 1**. **Figure 1A** shows the original image sample with the liver region in the red dashed line, and **Figure 1B** shows the processed liver image sample with the tumor region in the shaded part in the red dashed line. The white image in **Figure 1C** is the tumor label of the original sample.

This paper is structured as follows: Section I introduces the work on liver tumor segmentation, Section II describes the research methods of this paper, Section III gives the experimental results, and Section IV presents the conclusion and summary.

## Materials and Methods

### Algorithm Flow

**Figure 2:**
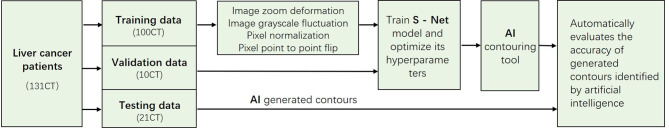
The 2017 LiTS public dataset with a total sample of 131 patients. In this study, we adaptively partitioned the entire sample into 100 training samples, 10 validation samples, and 21 test samples. The training samples are pre-processed, and the test samples are used to evaluate the segmentation effect of the network model.

The main process of the algorithm in this study included three stages: pre-processing, tumor segmentation, and post-processing **Figure 2**.

**Figure 3:**
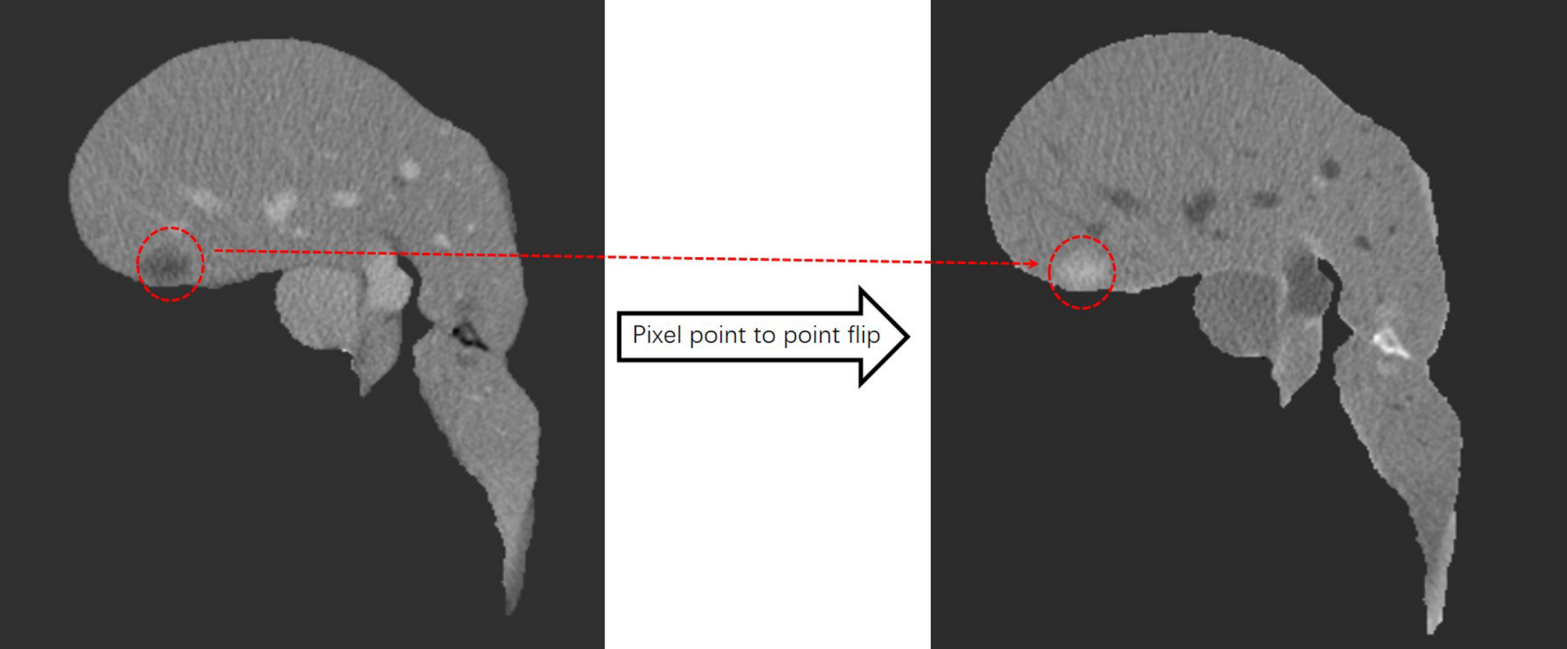
After pixel point-to-point flipping, the liver turns gray, the tumor turns white, and the area outside the liver is black.

In the pre-processing stage, we discussed basic processing means, such as image scaling deformation, grayscale floating, pixel normalization to eliminate overfitting, pixel flipping to change the image grayscale value, and point-to-point to flip the pixel grayscale. Image scaling deformation includes the rotation, mirroring, translation, and affine transformation of each layer of the CT image with its corresponding contour outline. Image grayscale float multiplies the grayscale values of all pixel points on the image by a random number between 0.8 and 1.2, and then superimposes a random number between -0.2 and 0.2. The pixel point-to-point flip first divides each pixel point of the foreground image by 255 to obtain a new pixel point and then subtracts the new pixel point by the value 1. This value is multiplied by 255 to achieve the function of the grayscale flip. The image after the point-to-point flip is shown in **Figure 3**.

**Figure 4:**
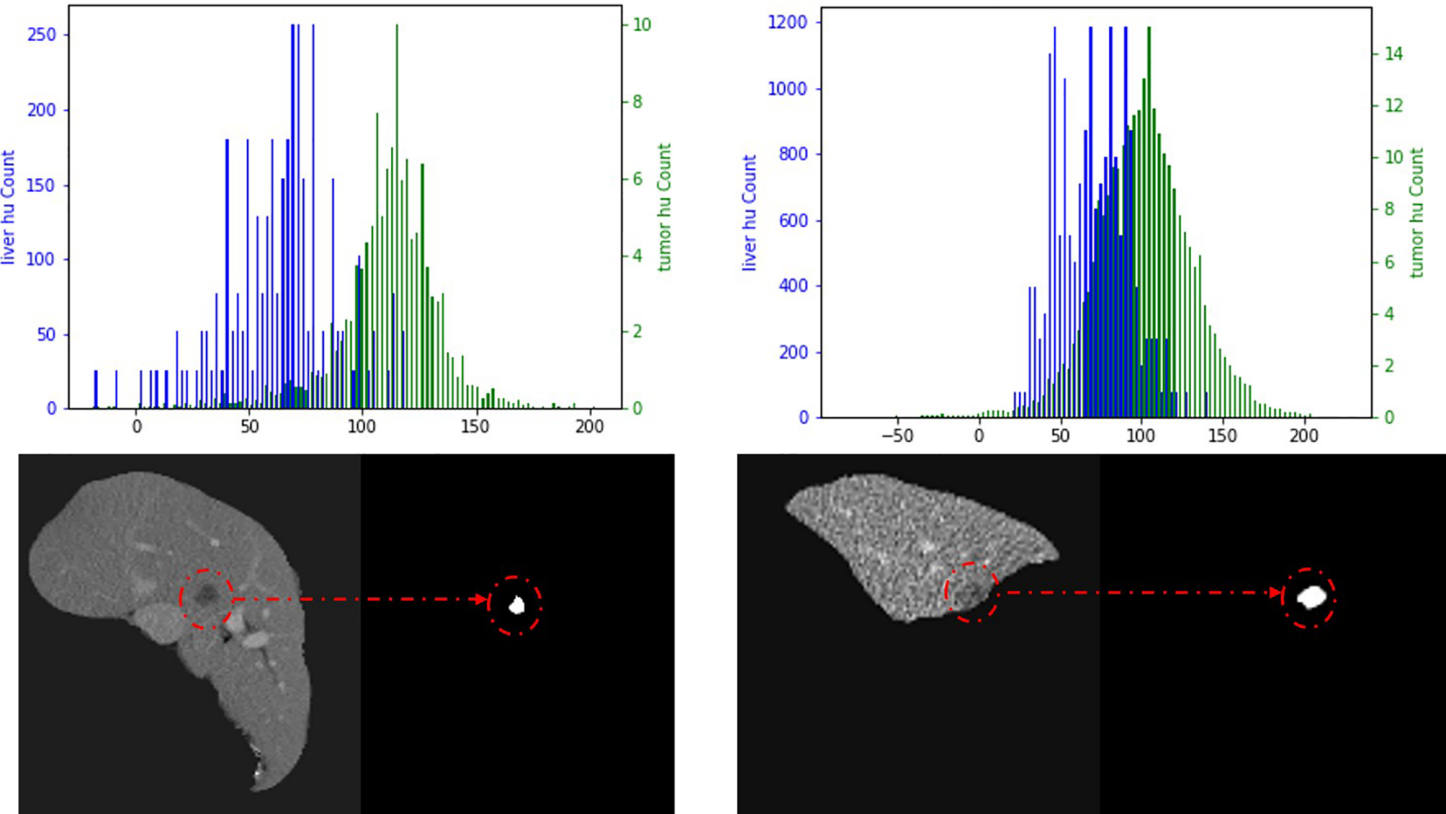
The left panel is a high-contrast section and the right panel is a low-contrast section. Blue is the liver HU value and green is the tumor HU value.

In 2D sections of some samples, the overlap of Hounsfield unit (HU) values between the liver and tumor leads to poor training and makes the network model misleading, especially during the learning process. As a result of this, we used the critical threshold method throughout this study to remove the sample cuts with low contrast to increase the learning ability of the network. **Figure 4** shows the two cuts with strong and low contrast, as well as the HU diagram. In the post-processing stage, we performed noise reduction through the closed operation.

### S-Net Network Architecture

**Figure 5:**
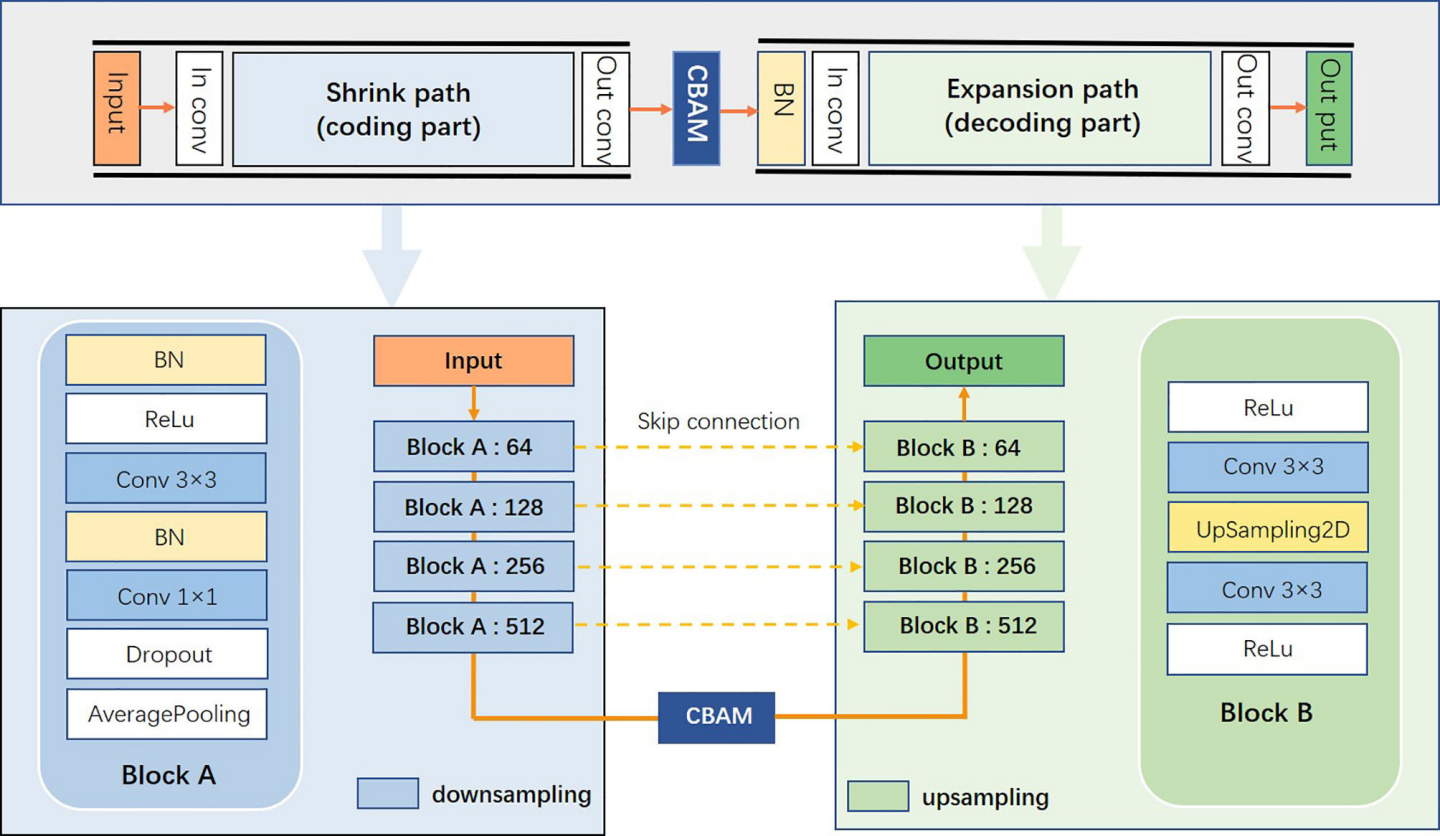
S-Net network architecture diagram.

This study proposed a novel convolutional neural network of S-Net based on 2D U-Net, as displayed in **Figure 5**. The architecture introduced an attention mechanism based on U-Net while using a typical encoding and decoding structure. In this structure, the left path is the contraction path (encoder) from top to bottom and the right path is the expansion path (decoder) from bottom to top. Because the target area of some samples was small, it was difficult to extract the semantic information in them. Therefore, we used small convolutional kernels and multiple batch processing for training. To extract deeper semantic information, the number of convolutional kernel channels of the contraction path was gradually increased. The feature map size gradually decreased in the down-sampling phase by reason of the pooling layer. In the up-sampling stage, the pooling layer was changed to an up-sampling layer because of the expansion path. This helped to recover the resolution of the original image. In addition, the number of convolutional kernel channels was gradually reduced to achieve end-to-end segmentation. At the intersection of contraction and expansion paths, we introduced a spatial attention mechanism and a channel attention mechanism to enable the network to encode longer-range semantic information in local features and to find correspondences between different channels. We introduced the long-hop connection between the layers of the contraction path and the expansion path so that the semantic information extracted in the contraction path was fused with the semantic information extracted in the expansion path.

**Figure 6:**
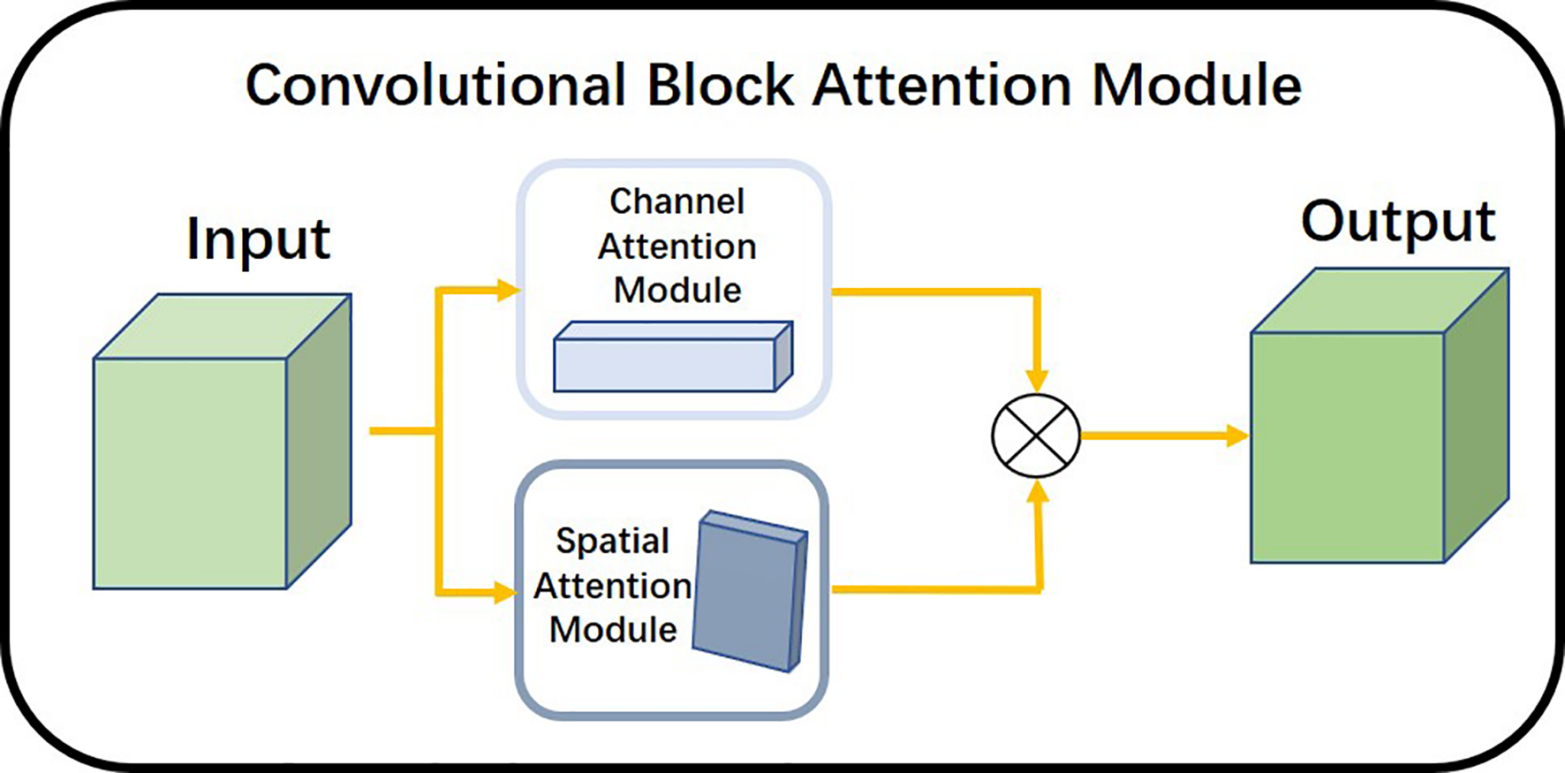
Overall architecture of the attention mechanism.

The Convolutional Attention Module is a simple and effective attention module for feed-forward convolutional neural networks. The overall architecture is shown in **Figure 6**. The attention module inferred attentional regions along two specific and mutually independent dimensions, multiplied the channel attention mechanism with the spatial attention mechanism, and adaptively optimized the local features. Because the attention mechanism architecture was small and lightweight, it could be seamlessly integrated into any network architecture and could be trained end-to-end along with neural networks.

**Figure 7:**
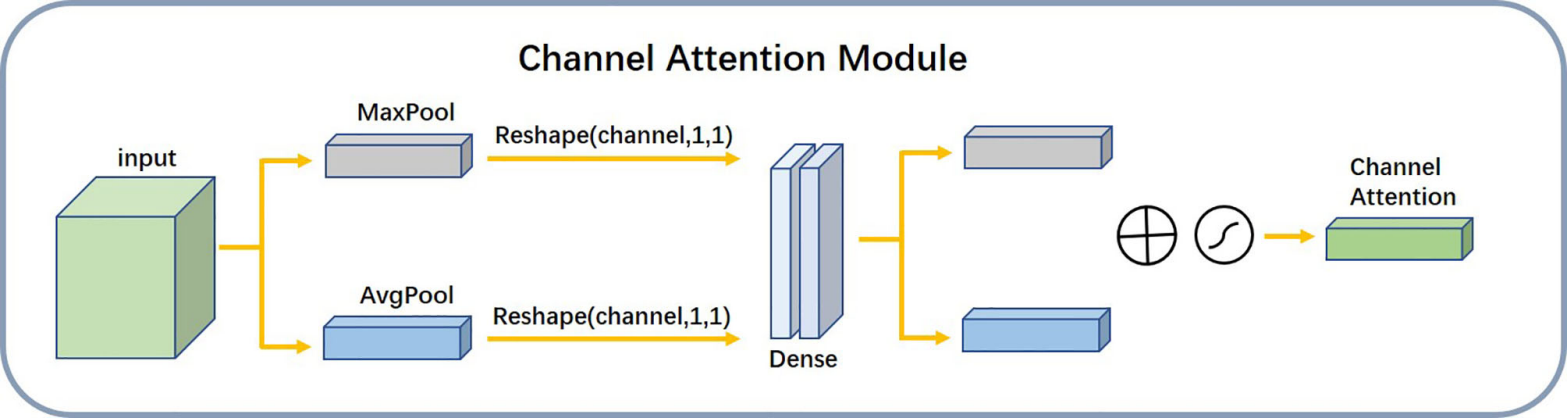
Structure diagram of channel attention module.

We used the channel attention module illustrated in **Figure 7** to find the dependencies between different channels and to enhance the dependent features. It focused mainly on the region of interest of the input image and compressed the spatial dimension of the input feature map. The module used the average pooling layer $F_{avg}^{c}$ and the maximum pooling layer $F_{max}^{c}$ to extract semantic information between channels. The shared network consisted of multiple layers of perceptrons. The workflow of the module is described as follows:

Maximum Pooling and Average Pooling Feature Maps: We used the maximum pooling layer to select the maximum value of the image region as the pooled value of the region. This eliminated nonextreme values and reduced the complexity of the upper-layer calculation. In addition, this layer could achieve translation invariance. The average pooling layer calculated the average value of the image region as the pooled value of the region. This could fade the combination of the relative positions between different features. Passing the pooled output through multiple layers of perceptrons: The multilayer perceptron played the role of a dimensional transformer. It converted high-dimensional information into low-dimensional information while preserving useful information. After a SoftMax function: The attentional mechanism was nonequivalent to the input of the overall sample. The channel attention module analyzed the weight of all input channels of a certain feature map and automatically selected the channels that need to be emphasized. The model set higher weights where necessary and smaller weights for those channels that were not emphasized. We used the SoftMax function to generate the probability of the importance of each channel.

**Figure 8:**
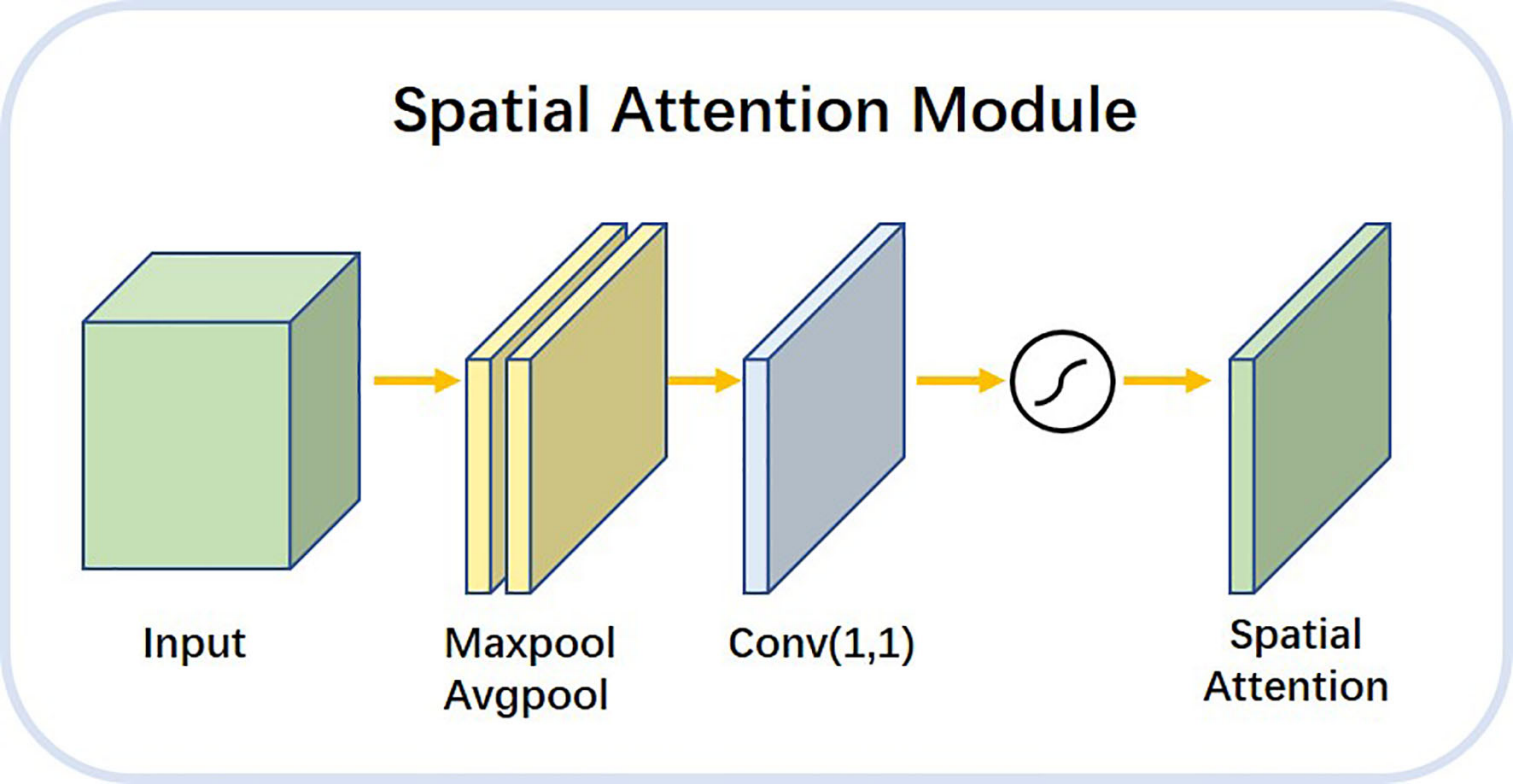
Architecture diagram of spatial attention module.

The spatial attention module shown in **Figure 8** allowed the network to encode a longer range of semantic information in local features. Unlike the channel attention module, spatial attention focuses on the “where” as the most informative piece of information and complements channel attention. We applied the average pooling and maximum pooling operations along the channel axes and concatenated the operations to produce valid feature descriptors. With these descriptors, the channel information was merged to produce two feature maps: $F_{avg}^{c},F_{max}^{S}$. The workflow of the spatial attention module is described as follows. maximum pooling and average pooling were done along the channel level to compress the image of N channels of HxW into a single channel of HxW. The 1x1 convolution layer was a linear combination of each pixel on different channels that retained the original planar structure of the feature map. It only changed the number of channels, thus achieving both up and down dimensional functions. The final attention map was normalized by the SoftMax function.

## Results

### Evaluation Index and Experimental Result

To demonstrate the effectiveness of the algorithm, we conducted experiments on the LITS dataset, in which all image data was collected from academic and clinical institutions worldwide, including the data of 131 liver cancer patients. The number of layers per CT scan varied between 42 and 1026 for each sample, whereas the pixel size of each CT layer was 512 × 512 pixels. The number of liver tumors in each sample ranged from 0 to 75 and the size of tumors ranged from 38*mm* ^3^ to 349 *mm* ^3^.

To evaluate the effectiveness of S-Net, researchers calculated the overlap measure according to the evaluation of LITS dataset, including Dice Global (DG) score, Dice per Case (DC) score, volumetric overlap error (VOE), average symmetric surface distance (ASSD), and root mean square error (RMSE). The Dice Global (DG) score is applied across all cases if they combine in a single volume, while the Dice per Case (DC) score refers to an average Dice score per volume. The mask labels provided by the LiTS dataset, 3DIRCADb dataset and doctors’ manual contours of Hubei Cancer Hospital dataset are defined as the gold standard. The Dice score can be formulated as:

$$Dice(A,B)=\frac{2\left|A\cap B\right|}{\left|A\right|+\left|B\right|}$$

In this formula, A represents predicted results while B represents true annotations. The loss function, Loss, was calculated using the formula:

Loss=1−Dice(A,B)

The DC, DG, VOE, ASSD and RMSE for this study were found to be 0.613, 0.755, 0.413, 1.186, 1.703, respectively. These values of this paper’s network architecture and three different network architectures (U-Net, DenseNet, and ResNet) were measured separately using the LiTS dataset as shown in **Table 1**. The models in the experiments all used 2D convolutional neural networks. When compared with the U-Net network, the DC value is improved by 0.099, the DG value was improved by 0.112, the VOE value is declined by 0.12, the RMSE is declined by 0.574. When compared with the three networks, these values were also found to show significant improvement. These values of different networks were also improved after applying the post-processing method.

**Table 1:** Liver tumor segmentation results by S-Net, and U-Net, DenseNet and ResNet.

Model	Dice per case (DC)	Dice global (DG)	volumetric overlap error (VOE)	average symmetric surface distance (ASSD)	root mean square error (RMSE)
U-Net (10)	0.514	0.643	0.533	1.763	2.378
U-Net and post-processing	0.525	0.651	0.527	1.759	2.372
DenseNet (22)	0.504	0.612	0.557	1.862	2.765
DenseNet and post-processing	0.513	0.623	0.546	1.831	2.755
ResNet (23)	0.503	0.631	0.431	1.279	2.076
ResNet and post-processing	0.512	0.642	0.427	1.254	2.059
S-Net	0.613	0.755	0.413	1.186	1.804
S-Net and post-processing	**0.647**	**0.761**	**0.409**	**1.177**	**1.801**

Compared with U-Net, DenseNet and ResNet, the S-Net proposed in this paper has a higher DC value and DG value, lower VOE value, RVD value, ASSD value and RMSE value. The meaning of the bold values are best results.

### Experimental Details and Parameter Settings

In this study, we adaptively partitioned all samples into 100 training samples, 10 validation samples, and tested our trained model from the LITS dataset on 3DIRCADb dataset and Hubei Cancer Hospital dataset. The 3DIRCADb datasets are composed of 20 CT scans, where 15 cases have hepatic tumors in the liver. The Hubei Cancer Hospital datasets included 20 enhanced CT scans of hepatic carcinoma with contrast from radiology department of Hubei Cancer Hospital. The auto-delineation results of this study used by Hubei Cancer Hospital dataset are shown in **Figure 11**. The training samples were pre-processed and the test samples were used to evaluate the segmentation effect of the network architecture.

**Figure 9:**
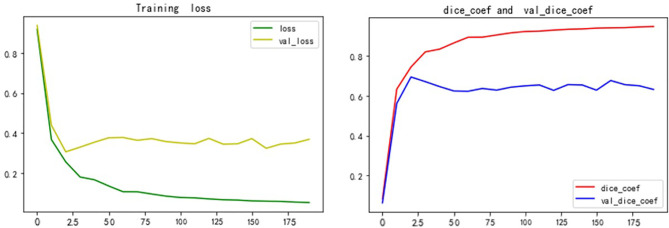
Dice and Loss values for training and verification.

We built the network architecture using Keras, with an NVIDIA Tesla P100 graphics card, and trained the network using a momentum gradient optimizer. We found initial learning rate to be 0.01, When the loss rate of the verification set did not decrease in three cycles, the learning rate was automatically reduced. A total of 200 cycles were trained. The set of weights with the highest Dice coefficients on the validation set was saved as the set of weights used for the testing phase. The activation function was a linear correction unit (RELU). The Dice and Loss values for training and validation are shown in **Figure 9**.

### Auto-Delineation Results

**Figure 10:**
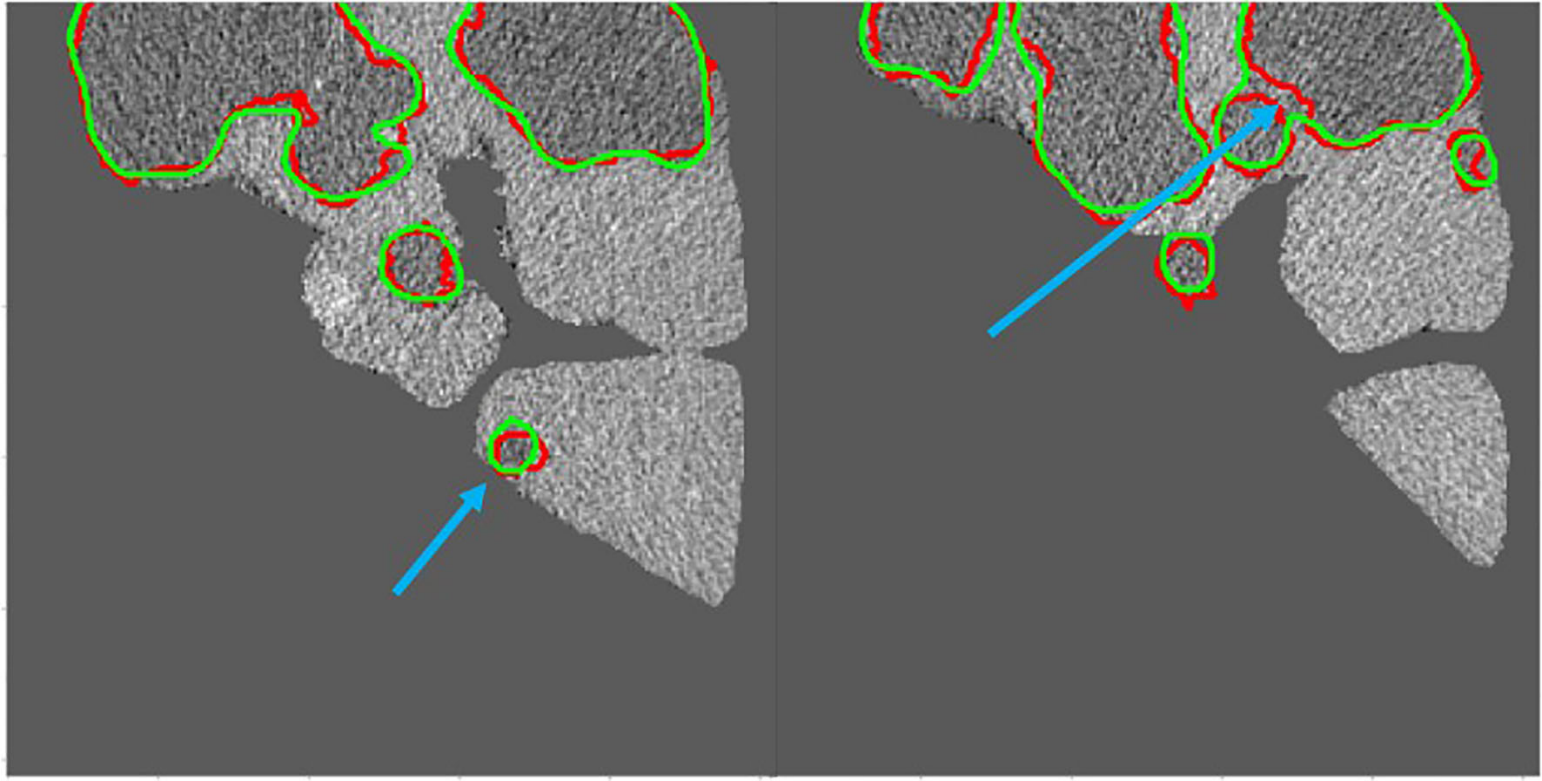
Green contour shows the predicted results by S-Net models, while red shows the gold standard. The small and multiple lesions, marked by the blue arrows.

**Figure 10** shows the 2D visualizations of the auto-segmented contours for selected two CT scans from LiTS dataset. Green lines represent predicted results by S-Net model, while the red ones are gold standard. As can be seen from the figures, the auto-segmentations were close to the gold standard delineations, especially for the large liver lesions. The small and multiple lesions, marked by the blue arrows in figures, the overlapped regions slightly reduced. Therefore, the S-Net model could well segment the liver and liver tumors, but as for small and multiple tumors, it still needs more attention to enhance.

**Figure 11:**
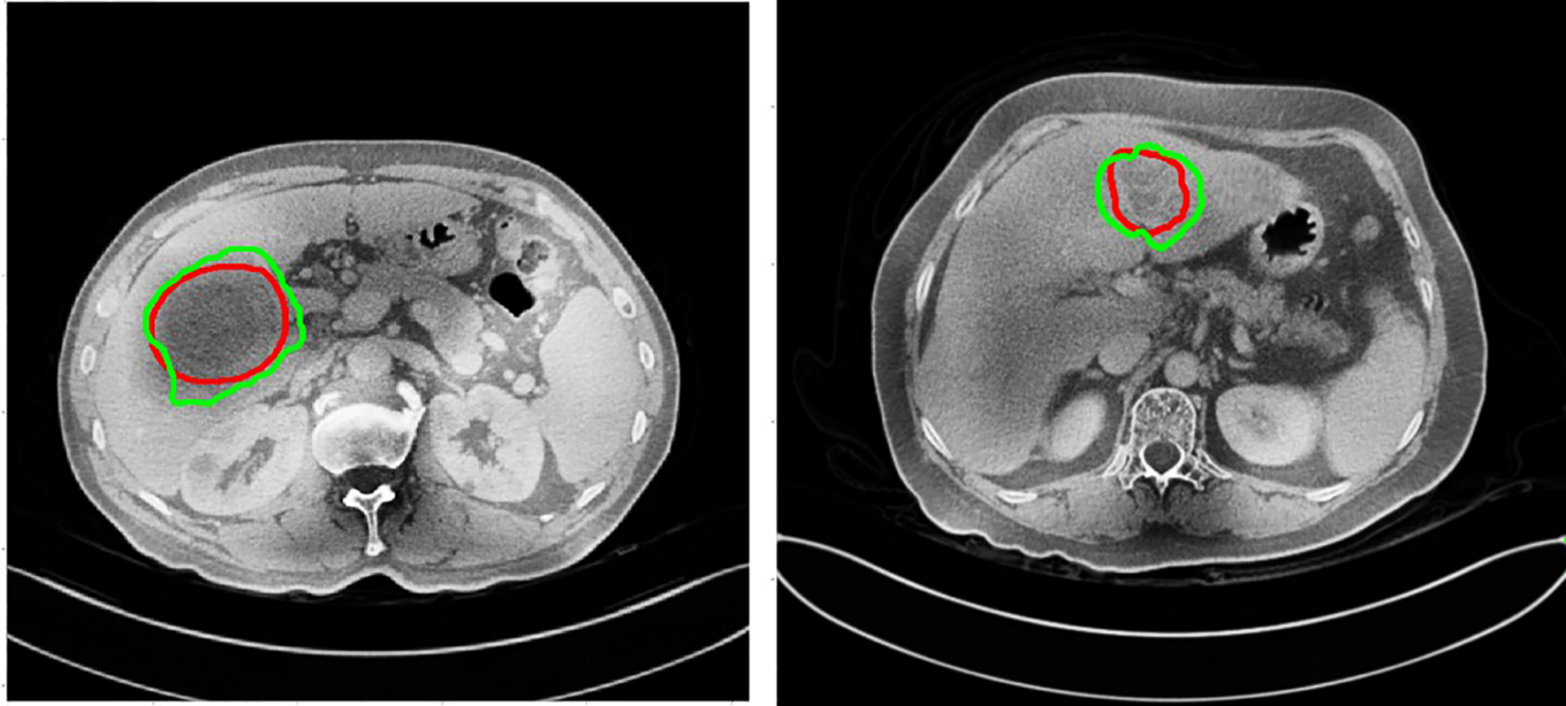
The auto-delineation results of this study used by Hubei Cancer Hospital dataset. Green contour shows automatic results, while red shows the gold standard.

## Discussion

In this study, researchers proposed a novel convolutional neural network called S-Net to auto-segmentate liver tumors. The evaluation metric DC, DG, VOE, ASSD and RMSE were found to be 0.613, 0.755, 0.413, 1.186 and 1.804 respectively. The novel network S-Net was able to outperform other networks like U-Net, DenseNet, and ResNet. In addition, we proposed a pre-processing method of pixel point-to-point flipping, which improved the contrast of the HU values of CT sections, made the network learn useful information more easily. Unlike existing FCN network architectures, this architecture had the following two features:

1. Ability to add spatial attention mechanism and channel attention mechanism between encoder and decoder. The spatial attention mechanism allowed the network to encode a longer range of semantic information in local features, whereas the channel attention mechanism found correspondences between different channels. In practice, the attention mechanism allowed the network to fully focus on the learning area, which greatly improved the accuracy of segmentation.
2. Long-hop connections increased the fusion rate of spatial feature information in the network, which could aid in the transfer of different spatial feature information from layer to layer.

On the basis of the 2017 LITS dataset, which tested the learning ability of the network, we concluded that the S-Net used in this study improved DC and DC values when compared with the U-Net network. DG and DC values were improved by 0.112 in and 0.117 in, respectively. When compared with cutting-edge algorithms like the cascaded FCN architecture proposed by Bellver et al. (24), these values improved by 0.015 and 0.023, respectively. When compared with the convolutional neural network incorporating spatial feature information proposed by Liu et al. (21), the DC value was improved by 0.021. The proposed network has a higher Dice value compared with Kaluva (25) and Pandey (26) et al., who added a residual structure to the conventional U-shaped structure to segment the tumor. The main reason for the improvement of segmentation accuracy is that this architecture adopted a canonical code-decode structure. It also integrated an attention mechanism based on the original U-Net network and used small, convoluted kernels to extract small amounts of semantic information. This architecture used a modular approach to gradually increase the number of convoluted kernel channels that extract deeper semantic information, thus improving the accuracy of segmentation. These results are shown in **Table 2**.

**Table 2:** Liver tumor segmentation results compared with other methods on the LiTS test dataset.

Model	Dice per case (DC)	Dice global (DG)	volumetric overlap error (VOE)	average symmetric surface distance (ASSD)	root mean square error (RMSE)
Chlebus et al. (14)	0.580	—	—	—	—
Song et al. (27)	0.569	0.751	0.437	1.702	—
Kaluva et al. (25)	0.492	0.625	**0.411**	1.441	
Pandey et al. (26)	0.587	—	—	—	—
Bi et al. (26)	0.500	—	—	—	—
T Liu al (28).	0.592	0.764	0.416	1.585	—
Li et al. (11)	**0.722**	**0.824**	0.497	**0.529**	**1.111**
Our S-Net	0.613	0.755	0.413	1.186	1.804

The DC, DG, Precision of other methods are obtained from the LITS leaderboard. The S-Net achieves much better performance in the precision score of liver tumors. The bold digitals denote the best results.

We mainly selected the results based on 2D model (14, 25–27, 29), except for some 3D model results by Li and Liu (11, 28). The proposed S-Net with the fusion of spatial features and attention mechanisms, outperformed than other 2D models. Indeed, the LiTS Leaderboard currently shows many higher DC and DG scores than those in the manuscript, especially the highest DC and DG by user ‘liver_seg’ were 0.7990 and 0.8500 respectively. Although some of the results are very high, there are still some reasons why we do not choose to repeat them. Firstly, it remains difficult to give recommendations about the exact network design, since the number and order of CNN layers and other hyperparameters were rough ideas instead of strict, proven guidelines. Secondly, the use of 3D architectures outperformed than the 2D ones, but in clinical practice they were not widely implemented due to memory constraints. Thirdly, more standard contour datasets will improve the segmentation accuracy, because state-of-art methods highly benefit from larger training datasets.

To demonstrate the applicability of our method in clinical practice, in this study we tested our trained model from the LiTS datasets on the new 3DIRCADb and Hubei Cancer Hospital datasets as shown in **Table 3**. The auto-delineation results of this study used by Hubei Cancer Hospital dataset are shown in **Figure 11**. And it achieved the slightly decreased results on tumor segmentation, with 0.578 on DC, 0.706 on DG, 0.576 on VOE and 1.673 on ASSD in 3DIRCADb dataset, with 0.527 on DC, 0.654 on DG, 0.594 on VOE and 1.862 on ASSD in Hubei Cancer Hospital dataset. The auto-segmentation results of 3DIRCADb dataset and Hubei Cancer Hospital dataset indicated the robustness of S-Net. Although the evaluation metric slightly decreased, it can effectively improve the effect of tumor recognition in CT images and could be applied to assist doctors in clinical treatment. If more datasets from different clinical centers are added for training, it is believed that the accuracy could be further increased.

**Table 3:** Tested our trained model from the LITS dataset on 3D-IRCADb dataset and Hubei Cancer Hospital dataset.

Dataset	Dice per case (DC)	Dice global (DG)	volumetric overlap error (VOE)	average symmetric surface (ASSD)
**3D-IRCADb**	0.578	0.706	0.576	1.673
**Hubei Cancer Hospital**	0.527	0.654	0.594	1.862

**Figure 12:**
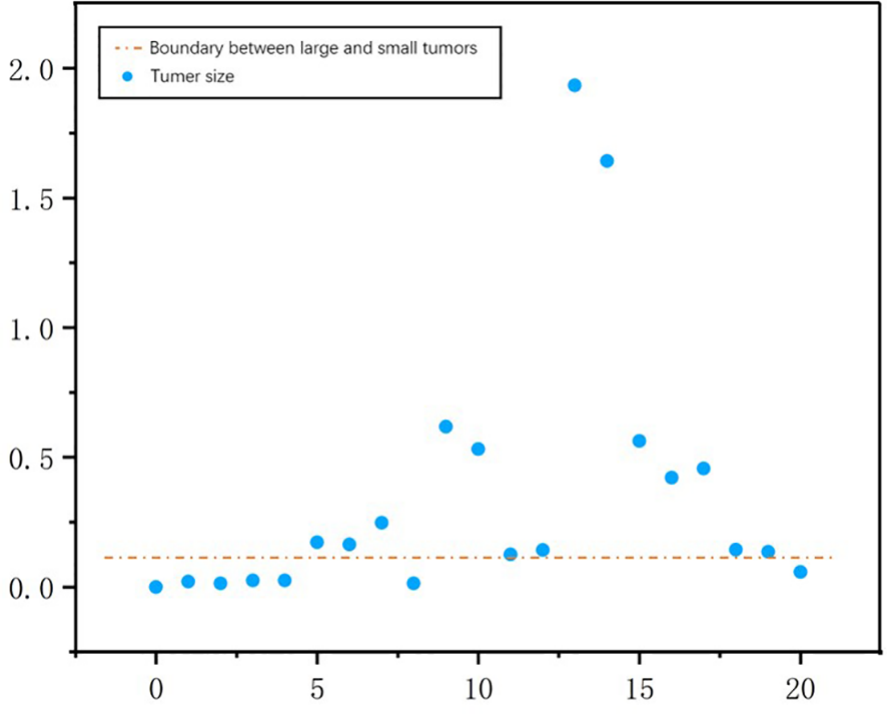
The number of tumor voxels per patient in the test dataset. The orange line was used to distinguish between a large tumor group and a small tumor group.

A threshold of 0.2 was used to distinguish large tumors from small ones. We calculated tumor size by aggregating the tumor voxels in each real CT image. We did this to further understand the performance metrics of the network and to analyze the accuracy of the S-Net architecture in identifying the tumor size of different patients. The voxel values of the 21 CT tumors in the test set are shown in **Figure 12**. We found the tumor sizes in this data set to be widely variable. To facilitate experimental development, the data set was partitioned into a large tumor group and a small tumor group. This was determined by the orange line. We have used Baseline values to shows the effectiveness of our network in small and large tumors, Baseline is the 2D U-Net **Table 4**. The DC value and DG value of the two sample groups were tested separately. These results are shown in **Table 4**. From **Table 4**, It can be clearly observed that the large tumor achieves 0.0469 (Dice per case) accuracy improvements while the score for the small-tumor group is slightly advanced, with 0.0353 (Dice per case). From the comparison, we claim that the main reasons for the improve in Dice value is by adopting adaptive attention convolutional neural network, which can notice different dimension of semantic information. As a result, the accuracy of segment large tumor will be improved considerably. But semantic information of small tumors are more difficult to extract, so segmentation for small tumors have limited improvement. This is because many small lesions only occupying a few voxels, and it’s difficult to distinguish the surrounding pixels in the lesion border. In addition, the difference in the HU value of liver and tumor may affect the segmentation accuracy.

**Table 4:** The effectiveness of our network to small and large tumors segmentations.

	Small tumors		large tumors	
	Dice per case	Dice global	Dice per case	Dice global
Baseline	0.2613	0.2917	0.7279	0.7548
S-Net	0.3082	0.3246	0.7632	0.7819

As can be seen from the table. The segmentation accuracy of large tumors are better than that of small tumors. Baseline is the 2D U-Net with trained model.

To tackle with the small and multiple liver tumor segmentation problem, several methods were proposed. Li et al. used perceptual generative adversarial networks (GANs) to generate super-resolved representation for small object by revealing the intrinsic structural correlations between small and large objects (30). Kamnitsas et al. proposed a multi-scale 3D CNN with fully connected CRF for small brain lesion segmentation (31). Some post-processing methods utilize a custom criteria of removing lesions as noise if they have large variation between adjacent slices, because the size of lesions usually increase/decrease gradually with image slices up-and-down (32). A new loss function combined with Dice score and focal loss was better for segmenting small-volume structures such as optic nerves and chiasm (33). In summary, GANs, multi-scale representation, new loss function and custom post-processing methods may be the potential solution to overcome this challenging problem (34). This should be explored further in future studies.

## Conclusion

In this study, an automatic CT image segmentation method based on S-Net network architecture used to automatically segment liver tumors from CT images was proposed. This study focused on the attention mechanism and the fusion of semantic information at different spatial dimensions. In this research, experiments based on the LITS dataset demonstrated that the methods discussed in this paper could improve the effect of automatic tumor segmentation in CT images. A drawback is that this algorithm was not effective in segmenting small tumors and multiple tumors. Future research will focus on the problem of case segmentation of small tumors and multiple tumors along with application of deep learning to clinical adjuvant therapy.

## Data Availability Statement

The datasets presented in this study can be found in online repositories. The names of the repository/repositories and accession number(s) can be found below: Figshare. DOI: https://doi.org/10.6084/m9.figshare.14926695.v1.

## Ethics Statement

Written informed consent was obtained from the individual(s) for the publication of any potentially identifiable images or data included in this article.

## Author Contributions

WW proposed the research idea. SL and XX performed the coding algorithms and manuscript drafting work. YD helped with the data analysis. WW and BZ gave useful discussions and provided the research platform. All authors contributed to the article and approved the submitted version.

## Funding

This study was supported by the National Natural Science Foundation of China: 12075095 and 11704108, the Health Commission of Hubei Province scientific research project WJ2021M192.

## Conflict of Interest

The authors declare that the research was conducted in the absence of any commercial or financial relationships that could be construed as a potential conflict of interest.

## Publisher’s Note

All claims expressed in this article are solely those of the authors and do not necessarily represent those of their affiliated organizations, or those of the publisher, the editors and the reviewers. Any product that may be evaluated in this article, or claim that may be made by its manufacturer, is not guaranteed or endorsed by the publisher.
